# Experimental Study on Low-Shrinkage Concrete Mix Proportion for Post-Casting Belt of Full-Section Casting in Immersed Tube

**DOI:** 10.3390/ma18143315

**Published:** 2025-07-14

**Authors:** Bang-Yan Liang, Wen-Huo Sun, Chun-Lin Deng, Qian Hu, Yong-Hui Huang

**Affiliations:** 1 The Second Engineering Company of CCCC Fourth Harbor Engineering Co., Ltd., Guangzhou 510230, China; liangbangyan@ccccltd.cn (B.-Y.L.); sunwenhuo@ccccltd.cn (W.-H.S.); 2 CCCC Fourth Harbor Engineering Institute Co., Ltd., Guangzhou 510230, China; dengchunlin@ccccltd.cn; 3 Research Center of Wind Engineering and Engineering Vibration, Guangzhou University, Guangzhou 510006, China; huqian@e.gzhu.edu.cn

**Keywords:** immersed tunnel, full-section casting, calcium–magnesium composite expansive agent, post-casting belt, concrete cracking

## Abstract

Full-section interval casting technology was adopted for the integral immersed tube of the Chebei Immersed Tunnel. Field tests (Chebei Immersed Tunnel) were conducted to establish the time-dependent development of the concrete shrinkage strain of the full-section casting segments. And laboratory experiments were then carried out to investigate the influence of factors such as the reinforcement ratio and stress, expansive agent content and composition, fly ash content, and curing temperature and humidity on the expansive effect of calcium–magnesium composite expansive agents. Field tests revealed that casting segments exhibit initial expansion followed by shrinkage, reaching a final strain of 348 με (microstrain). Laboratory investigations demonstrated that reinforcement (20–30 MPa stress) in post-casting belts effectively restrains segments without compromising the performance of calcium–magnesium composite expansive agents. The optimal 5:3:2 ratio of CaO, MgO 90s, and MgO 200s agents controlled shrinkage strain within 80 με by combining CaO’s rapid early expansion with MgO’s sustained effect. Field validation confirmed the mix’s effectiveness in preventing cracking, with key findings: (1) fly ash content and curing conditions significantly influence expansive behavior, and (2) shrinkage development can be precisely regulated through agent composition adjustments.

## 1. Introduction

Immersed tunnels are in a phase of rapid development in China. Currently, the main types of immersed tunnels are traditional riverbed immersed tunnels represented by the Luntou–Bio Island Tunnel and Nanchang Honggu Tunnel [1,2,3], and cross-sea immersed tunnels represented by the Hong Kong–Zhuhai–Macao Bridge immersed tunnel [4,5,6]. The new technologies, processes, and equipment brought by projects such as the Shenzhen–Zhongshan Channel, Chebei Tunnel, West Huizhan Road Overpass Tunnel, Dalian Bay Subsea Tunnel, and Xiangyang Han River Immersed Tunnel under construction will push the technology of immersed tunnel to a new level [7,8]. The crack control of the immersed tunnel segments is a difficult and complex technical problem [9,10]. Scholars have conducted extensive research on the crack control of immersed tunnel segments, and the main technical means used include the following.

First is the post-casting belt technology. The immersed tunnel mainly adopts an integral immersed tube [11,12]. A post-casting belt with a width of approximately 1.5 m is set between two segments to reduce the risk of cracking caused by concrete hydration heat, drying shrinkage, etc. [13]. Post-casting belt construction is generally carried out when the concrete of adjacent segments is no less than 42 days old. The application of post-casting belt technology has effectively improved the problem of cracking in the integral immersed tube, but previous experience has shown that post-casting belts are more prone to leakage problems.

Second is full-section casting technology. Layered casting of the segment is prone to cracks at construction joints. The self-waterproofing of concrete is the foundation of waterproofing for immersed tunnels, and the appearance of temperature cracks should be minimized as much as possible. The Hong Kong–Zhuhai–Macao Bridge immersed tunnel segments use full-section casting technology, which effectively reduces the risk of concrete cracking at construction joints [14]. Currently, full-section casting technology is rarely applied to integral immersed tubes. The Xiangyang Immersed Tunnel project, which is under construction, uses full-section casting technology for the integral immersed tube for the first time [15].

Third is concrete mix design [16,17]. The concrete mix proportion is optimized to select key parameters such as concrete strength development, hydration heat, shrinkage creep, etc. The Luntou–Biodao Island immersed tunnel project optimized the concrete mix design through model tests and obtained low-heat, low-shrinkage concrete [9,18].

Fourth is concrete temperature control technology. The hydration heat temperature field of concrete is actively controlled through main temperature control measures (buried cooling water pipes, insulation measures, etc.) and curing processes [19,20]. The Ningbo Changhong immersed tunnel, Guangzhou Zhoutouzui Tunnel, and others have effectively reduced the occurrence of temperature cracks though the burial of cooling water pipes, application of insulation, and moisture-retaining measures.

**Figure 1:**
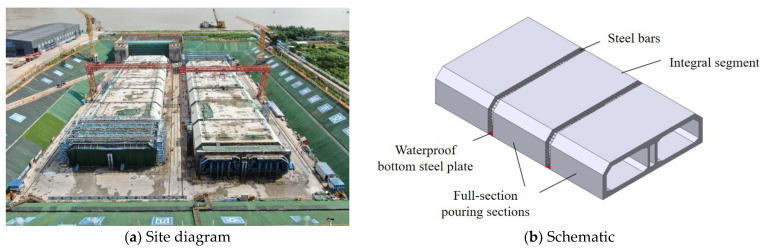
Full-section interval casting diagram of the Chebei Immersed Tunnel.

Drawing on the full-section casting technology from the Hong Kong–Zhuhai–Macao Bridge immersed tube tunnel [21], the integral immersed tube of the Chebei immersed tunnel adopts full-section interval casting technology. The integral immersed tube is divided into several segments, with post-casting belts set between them, where reinforcement can remain continuous and a waterproof steel plate runs throughout the tube. Both segments and post-casting belts use full-section casting technology, as illustrated in Figure 1. However, this process introduced a new issue: the impact of post-casting belt reinforcement on the full-section casting segments and the post-casting belt itself. Therefore, this paper, through field testing and laboratory experiments, investigated the development of shrinkage strain in full-section casting segments and the effect of post-casting belt reinforcement on the expansive performance of calcium–magnesium composite expansive agents. Furthermore, a concrete mix proportion for the post-casting belt, compatible with the shrinkage of full-section casting segments, was developed to prevent concrete cracking.

## 2. The Field Test Analysis for the Full-Section Casting Segment

### 2.1. Field Test

**Figure 2:**
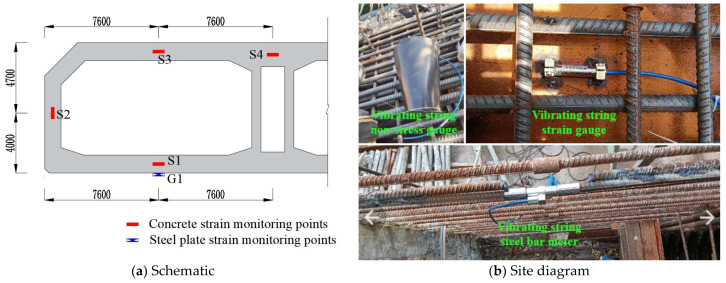
The layout of measurement points and the on-site installation diagram.

One section was arranged at the end of the full-section casting segment, adjacent to the post-casting belt. Four concrete strain measurement points named S1~S4 were arranged on the section, located at the center of each position, and vibrating string non-stress gauges were used to measure the concrete strain. And they could also measure the temperature of concrete. A vibrating string strain gauge named G1 was placed on the bottom steel plate to measure the steel strain below S1. Three vibrating string steel bar meters named A1~A3 were placed on the post-casting belt reinforcement to measure stress, and measurement points A1~A3 and S1~S3 correspond to each other, respectively. Figure 2 shows the layout of measurement points and the on-site installation diagram.

This study maintained curing temperatures between 20 °C and 40 °C with humidity above 80%. Retention was achieved via geotextile covering coupled with water spraying, curing shed, and an automated spray array system. In practical engineering, a dedicated curing system for immersed tunnel segments was developed, incorporating curing sheds and spray systems. The system will establish critical thresholds for temperature and humidity alarms. Through the continuous monitoring of the hygrothermal variation patterns both within the segment and in the curing environment, it will implement adaptive measures to regulate environmental conditions by either increasing or decreasing temperature and humidity levels as required.

### 2.2. Field Test Data Analysis

**Figure 3:**
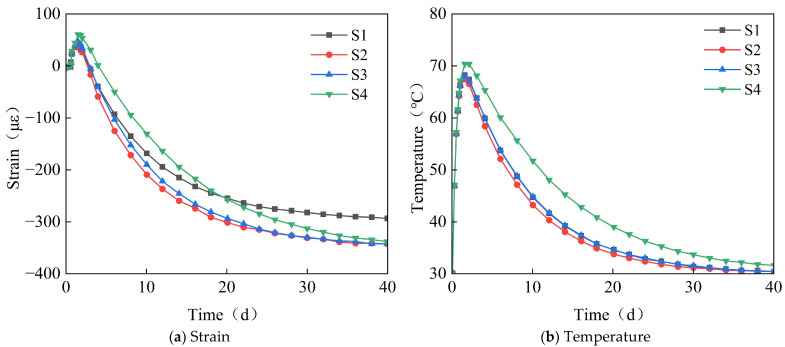
The time-variant curve diagrams of strain and temperature at measurement points S1–S4.

Figure 3 shows the time-variant pattern of the strain and temperature at measurement points S1−S4 for the full-section casting segment. Positive values indicate volumetric expansion (or temperature rise), while negative values indicate volumetric contraction (or temperature drop). As can be seen from Figure 3, during the early stages of concrete casting, the concrete expands due to the increase in hydration heat, reaching a maximum temperature of approximately 70 °C. This temperature increase causes the segment to expand, but since the concrete is in a fluid-plastic state currently, the expansion is not significant, with a maximum strain of 65 με. As time progresses, under the combined effects of hydration heat dissipation and concrete drying shrinkage, the concrete contracts, with the contraction gradually increasing and stabilizing after 40 days, reaching a maximum strain of −348 με.

**Figure 4:**
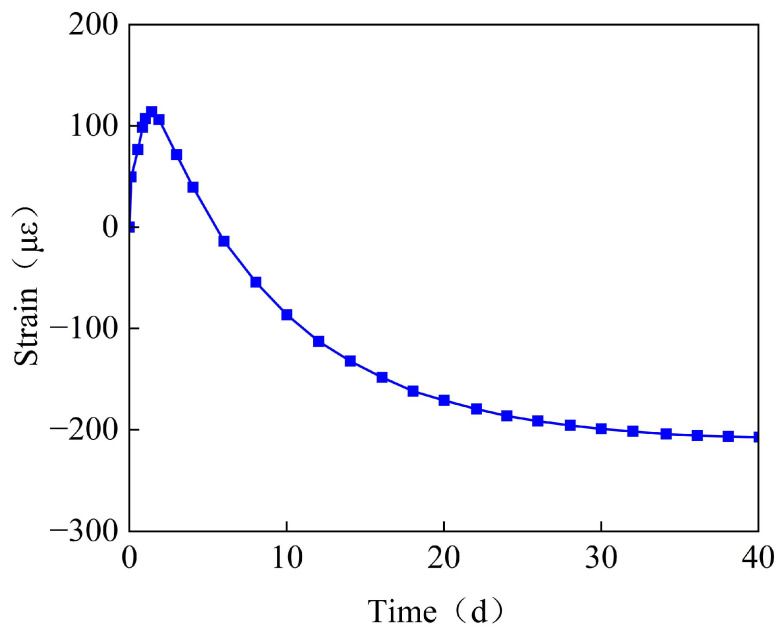
The time-variant curve diagram of the strain for the waterproof steel plate.

Figure 4 shows the time-variant pattern of the strain for the waterproof steel plate at measurement point G1. As can be seen from Figure 3, during the early stages of concrete casting, the waterproof steel plate expands, with a maximum strain of 126 με. Due to concrete being in a fluid-plastic state, the expansion of the waterproof steel plate is greater than that of the concrete. Subsequently, as the concrete strength increases, the waterproof steel plate and the concrete form an integral whole. Under the combined effects of hydration heat dissipation and concrete drying shrinkage, they contract together, with the contraction gradually increasing and stabilizing after 40 days. The maximum strain of the bottom steel plate is approximately −200 με.

**Figure 5:**
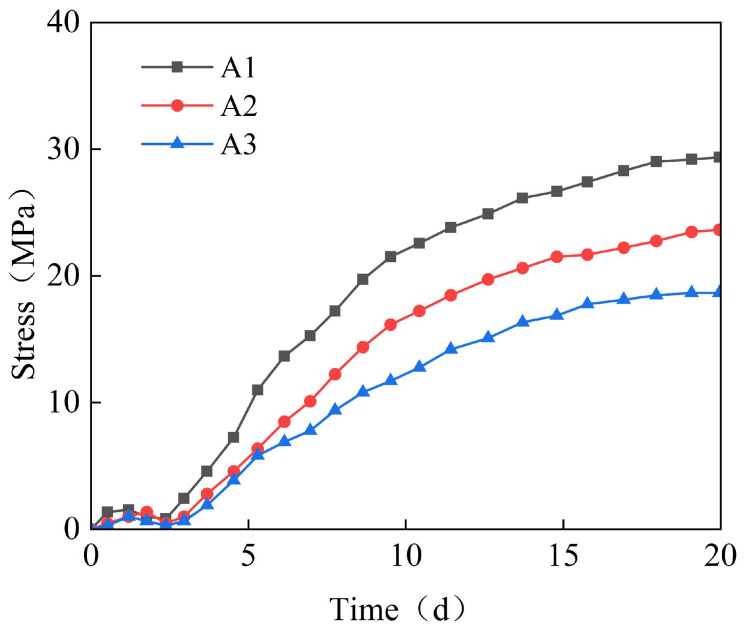
The time-variant curve diagram of the stress for the post-casting belt reinforcements.

The Chebei immersed tunnel adopts the casting method with the continuous post-casting belt reinforcements for the first time. The post-casting belt reinforcements exert a restraining effect on the full-section casting segment. Figure 5 shows the time-variant pattern of stress for the post-casting belt reinforcements at measurement points A1–A3. As can be seen from Figure 5, with the increase in concrete shrinkage, the stress of the reinforcements gradually increases, reaching a maximum stress of 28 MPa, located at the side wall.

## 3. The Laboratory Test Analysis for the Post-Casting Belt Concrete

### 3.1. Laboratory Test

Preventing the occurrence of cracks in post-casting belt concrete is a difficult task. By designing the concrete mix proportion and adopting appropriate curing measures, the concrete shrinkage can be controlled to meet the requirements for crack control. This paper proposes a strategy for regulating the volume of post-casting belt concrete. Based on field tests, the strain development pattern of full-section casting segment was obtained, which is used as the regulation target for designing the concrete mix proportion of the post-casting belt concrete.

**Figure 6:**
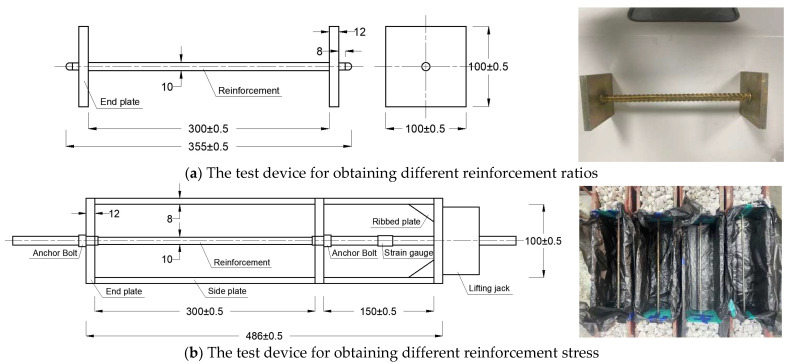
The test device for obtaining different reinforcement ratios or stresses.

**Figure 7:**
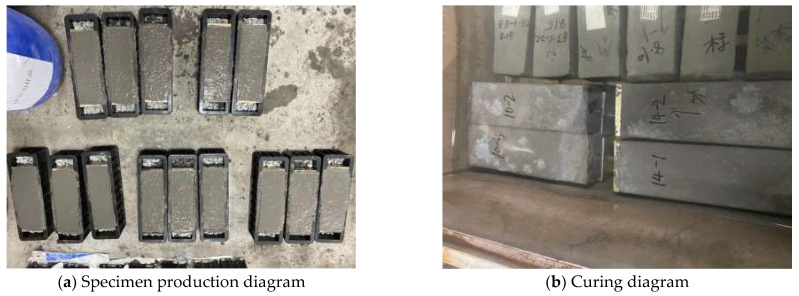
The specimen production and curing diagrams.

Through laboratory tests, the influence of factors such as the reinforcement ratio and stress of the post-casting belt, content and composition of the expansive agent, fly ash content, curing temperature, and humidity on the expansion effect of the calcium–magnesium composite expansive agent was investigated to support the optimal design of the concrete mix proportion. Eight experimental groups with 41 specimens were set up, with each specimen measuring 100 mm × 100 mm × 300 mm. To simulate the impact of the reinforcement ratio and stress on the expansive effect, a test device was designed, as shown in Figure 6. Different reinforcement ratios were obtained by adjusting the diameter of the steel bars, and various steel stresses were achieved by applying axial forces to the bars using a jack. C5M32 served as the baseline specimen, with its concrete mix proportion shown in Table 1. The mix proportions of the other specimens were adjusted according to the experimental objectives, as presented in Table 2. The diagrams of specimens are shown in Figure 7.

### 3.2. Laboratory Test Data Analysis

**Figure 8:**
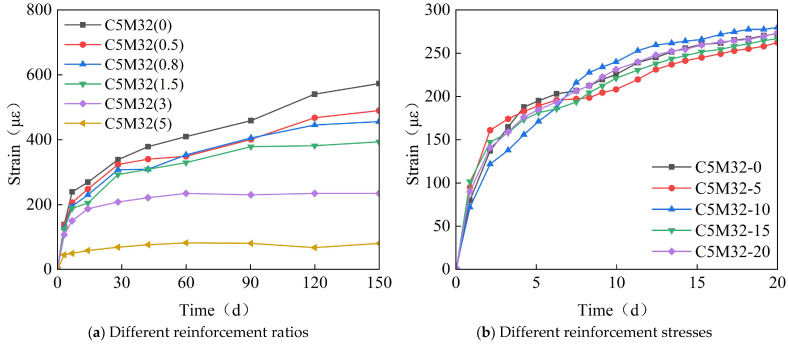
The time-variant curve diagram of the strain with different reinforcement ratios or stresses.

Figure 8 shows the time-variant pattern of the specimens’ strain with different reinforcement ratios or stresses. As seen in Figure 8a, the reinforcements have a restraining effect on the specimens’ strain. And the higher the reinforcement ratio, the greater the restraint. Due to the reinforcement ratio of the immersed tube generally being less than 1.5%, this restraint reduces concrete shrinkage strain by about 30%. Figure 8b indicates that there is no significant correlation between the reinforcement stress and the specimens’ strain, suggesting that the reinforcement stress does not affect the expansion effect of the calcium–magnesium composite expansive agent.

**Figure 9:**
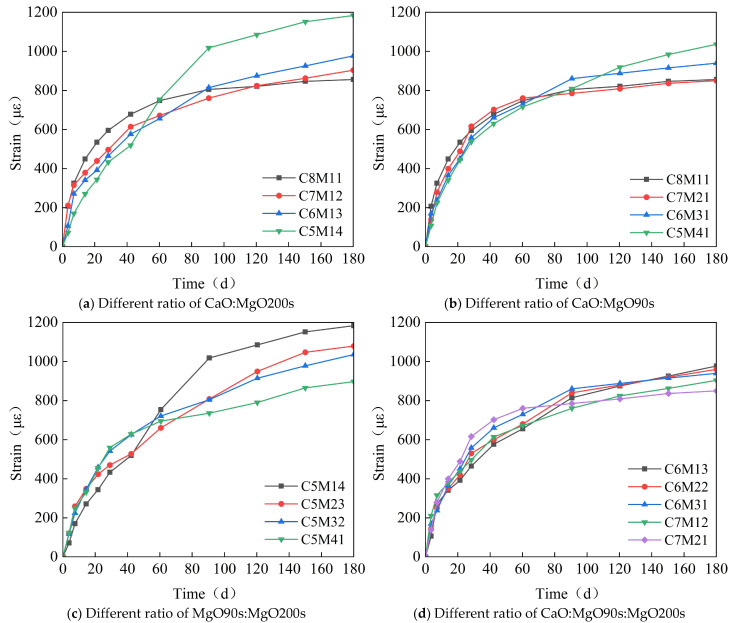
The time-variant curve diagram of the strain with different composition contents.

Figure 9 illustrates the time-dependent evolution of concrete strain under varying compositions of calcium–magnesium composite expansive agents, with subfigures (a) to (d) representing different CaO/MgO ratio gradients and MgO reactivity grades. The strain curves collectively demonstrate three distinct phases of development, rapid initial expansion (0–40 d), decelerated growth (40–100 d), and stabilization (>100 d), reflecting the dynamic interplay between hydration-driven expansion and drying shrinkage.

In Figure 9a,b, specimens with higher CaO content (e.g., C8M11 in Figure 9a) exhibit accelerated early-stage expansion, reaching 600–700 με within 40 days, followed by rapid stabilization. This phenomenon is attributed to the quick hydration of CaO, which generates ettringite and Ca (OH)_2_ crystals to fill capillary pores in the early curing period. Conversely, as MgO content increases (e.g., C5M14 in Figure 9a and C5M41 in Figure 9b), the strain curve shifts toward delayed expansion: the growth rate becomes significant after 40 days, with final strains exceeding 1000 με at 180 days. This indicates that MgO, particularly low reactivity grades, provides sustained expansive potential to counteract long-term shrinkage, consistent with its slower hydration kinetics and higher hydroxide gel formation efficiency.

The influence of MgO reactivity is further clarified in Figure 9c,d. Specimens incorporating MgO 200s (e.g., C5M41 in Figure 9c and C7M21 in Figure 9d) achieve approximately 5% higher ultimate strain (1150–1200 με) compared to those with MgO 90s (e.g., C5M14 in Figure 9c and C7M12 in Figure 9d). This performance difference arises from the finer particle size and higher specific surface area of MgO 200s, which prolongs hydration duration and enhances expansive agent utilization. Notably, the C7M21 mix in Figure 9d exhibits a distinct secondary expansion peak at 60–80 days, confirming the synergistic effect of CaO (early expansion) and MgO 200s (late expansion) in compensating shrinkage across the entire curing period.

Collectively, these results validate the tunability of shrinkage strain through compositional adjustment of calcium–magnesium expansive agents. CaO dominates early expansion to resist plastic shrinkage, while MgO (especially MgO 200s) governs long-term dimensional stability. By optimizing the CaO:MgO 90s:MgO 200s ratio (e.g., 5:3:2 as observed in high-performance mixes), concrete strain can be precisely regulated to achieve the target shrinkage control (<80 με) required for post-casting belt applications in immersed tunnel construction.

**Figure 10:**
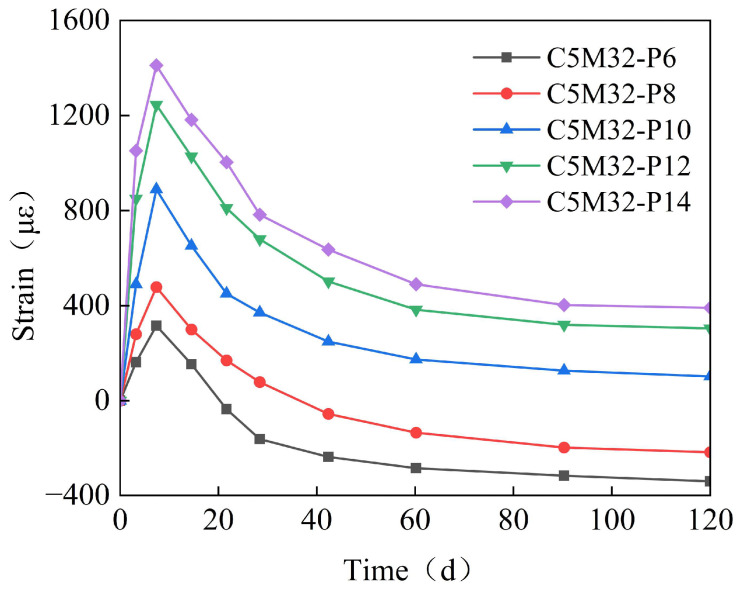
The time-variant curve diagram of the strain with different expansive agent contents.

Figure 10 shows the time-variant pattern of the specimens’ strain with different expansive agent contents. For the first 7 days, the specimens were cured in water at 40 °C to promote rapid expansion. Subsequently, they were dried in air with 60% humidity to induce shrinkage, allowing us to investigate whether the expansion from the expansive agent could counteract the concrete shrinkage. As shown in Figure 10, a higher expansive agent content results in greater expansion. When the content is less than 8%, the expansion is smaller than the concrete shrinkage, leaving the concrete in a contracted state. Above 10%, the concrete experiences rapid and significant early expansion, which may lead to cracking. Considering both the expansion magnitude and rate, an expansive agent content of 8% is deemed appropriate.

**Figure 11:**
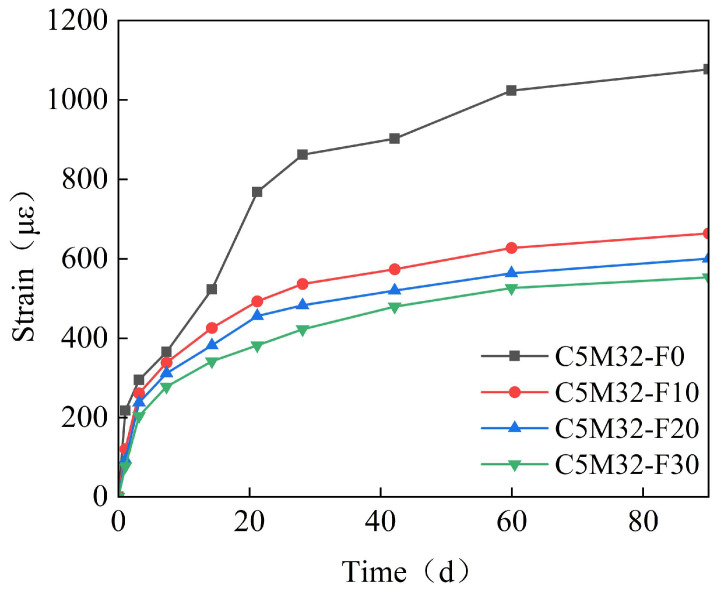
The time-variant curve diagram of the strain with different fly ash contents.

Figure 11 shows the time-variant pattern of the specimens strain with different fly ash contents. As shown in Figure 11, fly ash significantly inhibits the expansive effect of the calcium–magnesium composite expansive agent. As the fly ash content increases, the inhibition intensity increases, but the degree of inhibition gradually weakens. When the content reaches 30%, the expansion is reduced by 50%. It can be seen that the addition of fly ash acts as a buffer and leveler for the expansion of the calcium–magnesium composite expansive agent, which is a favorable factor for large-volume concrete structures.

**Figure 12:**
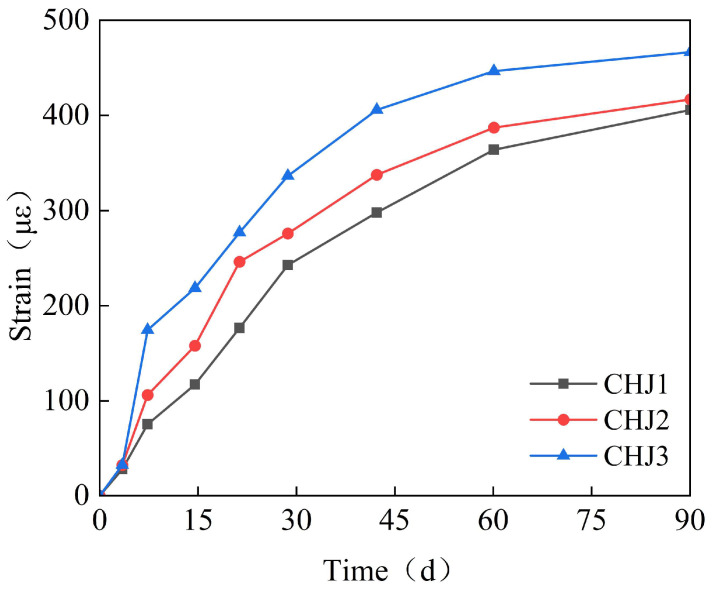
The time-variant curve diagram of the strain with different composition contents on the expansion effect.

Based on the above analysis, the expansive agent content for the Chebei Immersed Tunnel was selected as 8%, and the fly ash content as 20%. Three specimens, CHJ1, CHJ2, and CHJ3, were set up to further investigate the influence of expansive agent content on the expansion effect of the calcium–magnesium composite expansive agent. The specimens were cured in air with 85% humidity. The test results are shown in Figure 12. As seen in Figure 12, CHJ1 achieved an expansion of 400 με with a relatively slower early expansion rate, which is beneficial for early crack control in concrete. Therefore, the mix proportion of CHJ1 was ultimately chosen for the Chebei Immersed Tunnel.

**Figure 13:**
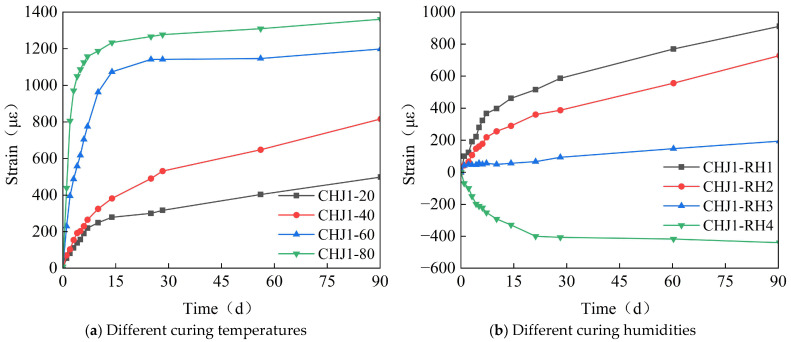
The time-variant curve diagram of the strain with different curing temperatures and humidities.

Figure 13 shows the time-variant pattern of the specimens’ strain with different curing temperatures and humidities. As seen in Figure 13a, a higher curing temperature results in a greater expansion of the calcium–magnesium composite expansive agent. To meet the deformation control requirement of 348 με over 40 days, a curing temperature range of 20–40 °C was selected. The curing temperature also affects the hydration heat temperature field of the concrete, so an appropriate curing temperature should be chosen in conjunction with the hydration heat temperature control requirements. As shown in Figure 13b, a higher curing humidity leads to a greater expansion of the calcium–magnesium composite expansive agent. When the curing humidity is below 70%, the expansive agent basically does not produce any expansion effect, and as the humidity continues to decrease, the expansive agent concrete shrinks. To meet the deformation control requirement of 348 με over 40 days, the curing humidity should be greater than 80%.

## 4. The Field Test Analysis for the Post-Casting Belt

**Figure 14:**
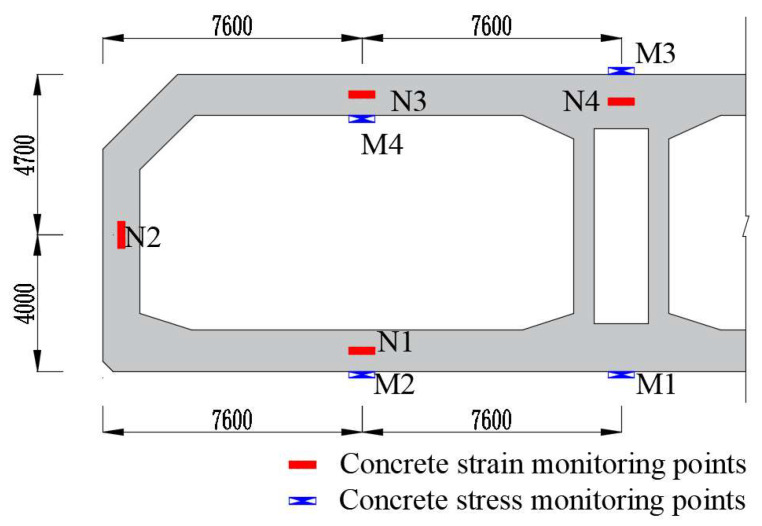
The layout diagram of measurement points for the post-casting belt.

To verify the effect of the calcium–magnesium composite expansive agent on regulating the deformation of post-poured belt concrete, tests were conducted on the shrinkage strain and stress of the concrete on-site. One section was arranged at the end of the post-casting belt, adjacent to the full-section casting segment. Four concrete strain measurement points named N1~N4 were arranged on the section, located at the center of each position, and vibrating string non-stress gauges were used to measure the concrete strain. Four vibrating string strain gauges named M1~M4 were placed on the surface of each position to measure the concrete strain. Figure 14 shows the layout diagram of measurement points.

**Figure 15:**
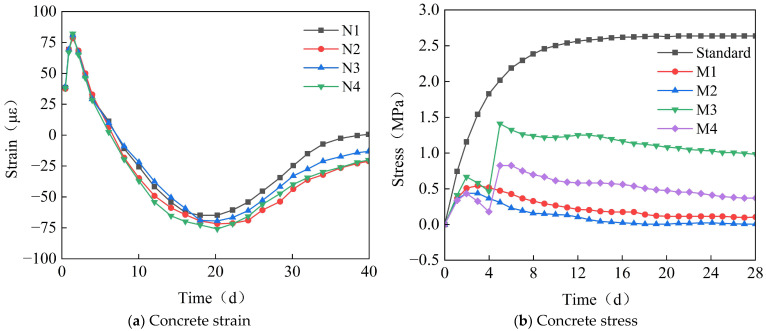
The time-variant curve diagram of the concrete strain or stress of the post-casting belt.

Figure 15 shows the time-variant pattern of the concrete strain or stress of the post-casting belt. As seen in Figure 15a, during the early stage of concrete casting, the concrete expands due to the combined effect of hydration heat and the expansive agent, with an expansion of about 80 με while in a fluid-plastic state. Subsequently, under the combined action of a temperature drop, concrete drying shrinkage, and expansion of the expansive agent, the concrete contracts. Compared to the full-section casting segment, the shrinkage is significantly reduced, with a maximum shrinkage strain of 75 με. As time progresses, the MgO expansive agent takes effect, reducing the concrete’s shrinkage strain to 20 με after 40 days. Thus, the calcium–magnesium composite expansive agent effectively regulates the volumetric deformation of the post-casting belt concrete. As shown in Figure 15b, the stress generated by concrete shrinkage is relatively small. The stress at measurement points M3 and M4 suddenly changes after the fourth day due to formwork removal and the effect of self-weight, causing variations in roof stress. During the early pouring stage, the concrete has low strength and can withstand limited tensile stress. Therefore, reducing the CaO content to prevent rapid concrete expansion is beneficial for crack control.

## 5. Conclusions

Through field testing and laboratory model experiments, this study developed a mix proportion for post-casting belt concrete based on the calcium–magnesium composite expansive agent, effectively preventing concrete cracking. The main conclusions are as follows:

(1) Under the combined effects of the hydration heat temperature field and concrete drying shrinkage, the full-casting section first expands and then contracts. Due to the low strength of early-stage concrete, the expansion is small, approximately 65 με. In the later stages, the concrete contracts, with a final convergence of about −348 με. This value is approximately 30% lower than that of conventional concrete (typically 500 με) and falls within the moderate-to-low shrinkage range. The observed strain aligns with data from major infrastructure projects, including the Hong Kong–Zhuhai–Macao Bridge immersed tubes (−310 με) and Shenzhen–Zhongshan Link tunnel segments (−380 με).
(2) The reinforcement exerts a restraining effect on the expansion of the calcium–magnesium composite expansive agent, with a reinforcement ratio of 1.5%, reducing the expansion by 30%. The ratio of 1.5% represents an upper-mid range value, while the Guangdong Provincial Standard for Steel-Shell Concrete Immersed Tunnels further recommends 0.8–1.8%. The observed reinforcement ratio aligns with data from major infrastructure projects, including the Hong Kong–Zhuhai–Macao Bridge immersed tubes (1.2–1.6%) and Shenzhen–Zhongshan Link tunnel segments (1.4–1.8%). The reinforcement in the post-casting belt exerts a restraining effect on the full-section casting segment, generating a stress of 20–30 MPa. However, the reinforcement stress does not affect the expansion effect of the calcium–magnesium composite expansive agent.
(3) CaO dominates early-stage expansion (0–40 days), achieving 600–700 με within 40 days, but exhibits negligible growth thereafter. In contrast, MgO provides sustained expansion: MgO 90s initiates significant expansion between 40 and 100 days, while MgO 200s extends this effect beyond 150 days, with specimens like C5M41 reaching 1200 με at 180 days.
(4) When the content is 8%, the optimal CaO: MgO 90s: MgO 200s ratio of 5:3:2 balances early and late expansion: it avoids excessive early stress via moderate 40-day strain (~500 με) while delivering 1150–1200 με ultimate strain—35% higher than CaO-only systems. Notably, MgO 200s outperforms MgO 90s by 5% in ultimate expansion and exhibits a distinctive “plateau growth” profile (60–120 d), mitigating thermal stress cracking.
(5) To ensure the control target of 348με for the calcium–magnesium composite expansive agent over 40 days, the curing temperature is selected to be between 20 and 40 °C, and the curing humidity is above 80%.
(6) The mix proportion of the post-casting belt based on the calcium–magnesium composite expansive agent developed in this study can control the convergent strain of the post-casting belt concrete to within 80 με, effectively preventing concrete cracking.

## Figures and Tables

**Table 1:** Concrete mix proportion of experimental specimen C5M32 (kg/m^3^).

Experimental Specimen Number	Cement	Fly Ash	Expansive Agent			Sand	Small Stone	Big Stone	Water	Water Reducer
			CaO	MgO 90s	MgO 200s					
C5M32	302	84	17	10.2	6.8	677	211	833	159.6	4.2

**Table 2:** Concrete mix proportions and curing methods for the experimental specimens (kg/m^3^).

Experimental Group			Reinforcement		Expansive Agent		Fly Ash Ratio	Curing Method
Experimental Objective	Number	Experimental Specimen Number	Ratio	Stress (MPa)	Ratio	CaO:MgO 90s:MgO 200s		
Study on the effect of different reinforcement ratios on the expansion effect.	1	C5M32(0)	0	0	8%	5:3:2	20%	Curing in water at 20 °C
	2	C5M32(0.5)	0.5%					
	3	C5M32(0.8)	0.8%					
	4	C5M32(1.5)	1.5%					
	5	C5M32(3)	3%					
	6	C5M32(5)	5%					
Study on the effect of different reinforcement stresses on the expansion effect.	7	C5M32-0	0.8%	0	8%	5:3:2	20%	Curing in water at 20 °C
	8	C5M32-5		5				
	9	C5M32-10		10				
	10	C5M32-15		15				
	11	C5M32-20		20				
Study on the effect of different expansive agent composition contents on the expansion effect.	12	C8M11	0.8%	0	8%	8:1:1	20%	Curing in water at 20 °C
	13	C7M12				7:1:2		
	14	C6M13				6:1:3		
	15	C5M14				5:1:4		
	16	C7M21				7:2:1		
	17	C6M22				6:2:2		
	18	C5M23				5:2:3		
	19	C6M31				6:3:1		
	20	C5M32				5:3:2		
	21	C5M41				5:4:1		
Study on the effect of different expansive agent contents on the expansion effect.	22	C5M32-P6	0.8%	0	6%	5:3:2	20%	The first 7 days involve water curing at 40 °C, followed by air curing with 60% humidity
	23	C5M32-P8			8%			
	24	C5M32-P10			10%			
	25	C5M32-P12			12%			
	26	C5M32-P14			14%			
Study on the effect of different fly ash contents on the expansion effect.	27	C5M32-F0	0.8%	0	6%	5:3:2	0	Curing in water at 40 °C
	28	C5M32-F10					10%	
	29	C5M32-F20					20%	
	30	C5M32-F30					30%	
Study on the effect of different composition contents on the expansion effect.	31	CHJ1	0.8%	0	8%	5:3:2	20%	Curing in air with 85% humidity at 30 °C
	32	CHJ2				6:3:1		
	33	CHJ3				7:2:1		
Study on the effect of different curing temperatures on the expansion effect.	34	CHJ1-20	0.8%	0	8%	5:3:2	20%	Curing in water at 20 °C
	35	CHJ1-40						Curing in water at 40 °C
	36	CHJ1-60						Curing in water at 60 °C
	37	CHJ1-80						Curing in water at 80 °C
Study on the effect of different curing humidities on the expansion effect.	38	CHJ1-RH1	0.8%	0	8%	5:3:2	20%	Curing in water at 30 °C
	39	CHJ1-RH2						Curing in air with 95% humidity at 30 °C
	40	CHJ1-RH3						Curing in air with 75% humidity at 30 °C
	41	CHJ1-RH4						Curing in air with 60% humidity at 30 °C

## Data Availability

The original contributions presented in this study are included in the article. Further inquiries can be directed to the corresponding author.
